# Effect of toxic trace element detoxification, body fat reduction following four-week intake of the Wellnessup diet: a three-arm, randomized clinical trial

**DOI:** 10.1186/s12986-020-00465-9

**Published:** 2020-06-22

**Authors:** Su-Jin Jung, Woo-Lim Kim, Byung-Hyun Park, Seung-Ok Lee, Soo-Wan Chae

**Affiliations:** 1 Clinical Trial Center for Functional Foods, Chonbuk National University Hospital, Jeonju, Jeonbuk 54907 South Korea; 2 Biomedical Research Institute, Chonbuk National University Hospital, Jeonju, Jeonbuk 54907 South Korea; 3 Department of Biochemistry and Molecular Biology, Chonbuk National University Medical School, Jeonju, Jeonbuk 54896 South Korea; 4 Division of Gastroenterology and Hepatology, Department of Internal Medicine, Chonbuk National University Medical School, Jeonju, Jeonbuk 54896 South Korea; 5 Department of Pharmacology, Chonbuk National University Medical School, 567 Baekje-daero, Deokjin, Jeonju, Jeonbuk 54896 South Korea

**Keywords:** Wellnessup diet, Toxic trace elements, Detoxification, Weight control, Calorie-restricted diet

## Abstract

**Background:**

Detox diet are known as a popular dieting strategies that helps toxins elimination and weight manage but there is very little clinical evidence. The Wellnessup diet (WD) used in the present study designed as a healthy meals based on organic plant based diets including various vegetables, fruits, whole grains, nuts and phytonutrients.

**Methods:**

To evaluate the effects of 4 week intake of the WD on toxic trace element detoxification, body fat reduction, and safety parameters. Forty-five women with body mass index (BMI) of 23.5–30 kg/m^2^ were recruited. Thirty of them were assigned 1:1 to the test group (WD, 15 subjects) and control group 1 (calorie-restricted diet, CRD, 15 subjects) in a single blind and randomized, and the remaining 15 subjects were assigned to control group 2 (maintaining regular diet, MRD). The primary outcome were toxic trace element levels in hair (29 types of heavy metals), and the secondary outcomes were changes in anthropometric and urinary organic acids.

**Results:**

The levels of four toxic trace elements in hair decreased in the WD group after the diet compared to before the diet. Ni, Rh, Sn, and Ga were significantly lower in the WD group than in the CRD or MRD group (*p* < 0.05). At the end of the trial, both WD and CRD groups had lower BMI, Waist Circumference(WC), Hip Circumference(HC) and WHR compared to the baseline values (*p* < 0.05). Compared to the WD group, the CRD group had a greater mean change (*p* < 0.05) from the baseline for weight loss (− 3.22 ± 0.48 kg vs − 1.88 ± 0.95 kg vs) and fat free mass (− 2.08 kg vs − 1.09 kg). The weight, BMI, body fat mass, fat free mass, WC, and HC of the CRD group were significantly decreased compared to the MRD (*p* < 0.05). No significant changes in any safety parameter were observed.

**Conclusions:**

Use of WD might have several beneficial effects and safety such as body fat reduction and improving some the element detoxification through caloric restriction but did not reducing body fat mass more than calorie-restricted diet.

**Trial registration:**

This study was registered at Clinical Research Information Service (CRIS) of Republic of Korea (KCT0003002).

## Introduction

Recently, with increasing industrialization, different chemicals and heavy metals have polluted the air and water, contaminating food and affecting health. Most of the environmental pollutants delivered through food are heavy metals and persistent organic pollutants (POPs). Chronic exposure to and accumulation of these pollutants have been linked to non-communicable diseases (NCDs) such as obesity, diabetes, cancer, cardiovascular diseases, and chronic respiratory disease [1–4]. Recent reports have indicated that accumulation of toxic trace elements in the body is related not only to metabolic disorders that cause overweight or obesity, but also to greater energy intake in people with obesity and diabetes. Furthermore, the diets of obese individuals often result in an imbalance of trace nutrients, and sufficient intake of vitamins and minerals has been emphasized in the management of obesity [5]. Detox dieting has become a popular strategy to promote toxin removal and weight loss, thus enhancing health and improving quality of life. However, detox diets are controversial, as some argue that there is a lack of scientific evidence for their health benefits, or that such diets may even be harmful [6–9]. Nevertheless, food-based nutrients have been studied for their ability to modulate metabolic pathways involved in detoxification processes. Several preliminary studies have demonstrated that food extracts and nutrients can regulate the transduction and eventual excretion of toxins [10–15]. Typically, detox diets are calorie-restricted diets consisting of a single fruits, vegetables, or beverages (tea, vinegar, lemon juice, salt water, or drinks mixed with micronutrients) [10–25]. The diet detox program known as the lemon diet and hypocaloric Mediterranean diet [16, 26] were a very low-calorie diet (LCD) that allows 500 to 1000 kcal per day and is effective in reducing body weight and fat; however, this dietary intervention is difficult to maintain and can lead to shortages of minerals, vitamins, and dietary fiber, as well as increased binge eating and stress [27, 28]. Moreover, while fasting or LCDs may allow weight loss, they can also lead to various health problems, such as malnutrition, muscle weakness, nervousness, headaches, dizziness, fatigue, gastrointestinal disturbances, and reduced quality of life [29, 30]. Several pre-clinical studies have reported the effects of detox diets [31–33], mostly with respect to detoxification, and some clinical studies have been published [10–16, 18–20, 22, 24–26].

In general, the effect of the detox diet on obesity was assessed for the weight loss effect in a short term period, but the biomarker was not measured for the detoxification effect of harmful elements. Furthremore, no scientific studies have investigated the effectiveness of nutritionally balanced detox diets for weight loss and toxin elimination. Moreover, studies comparing and evaluating the effects of a organic plant-based diet and conventional farming plant-based diet on weight loss and toxin elimination are insufficient.

Therefore, in this study, the WD was applied to minimize problems with existing detox diets. The diet of the WD consisted of organic ingredients produced by smart farms (pesticide-free and pollution-free). The Wellnessup diet (WD) was designed as a healthy meals based on organic plant based diets including various vegetables, fruits, whole grains, nuts and phytonutrients [34]. We performed a four-week, single blind, randomized, controlled pilot study to evaluate the efficacy and safety of the WD and CRD for toxic trace element detoxification and reduction of body fat in overweight young women.

## Materials and methods

This study was reviewed and approved by the Institutional Review Board (IRB) (CUH 2017–11-009) of Chonbuk National University Hospital (CUH). The entire human study was conducted in accordance with the provisions of the Helsinki Declaration and the standards for clinical trial management (IGCP), and the protocol was registered at Clinical Research Information Service of Republic of Korea (https://cris.nih.go.kr/cris/en/: board approval number: KCT0003002). This study was performed from January 2018 to March 2018 as a single-organization, randomized, parallel-group-controlled clinical study.

### Subjects

The subjects in this study were recruited from the Clinic Trial Center for Functional Foods (CTCF2) at CUH through advertising (brochures, posters) and the CUH website. Volunteers were evaluated for eligibility after providing written consent. Suitable subjects for this study were selected through screening tests such as medical interviews, medical examinations, and diagnostic medical examinations within four weeks (Day − 28 to Day − 1) of the baseline evaluation date (Day 0). To meet the criteria for selection, subjects needed to be women older than 19 and younger than 49 years at the time of the screening test, with body mass index (BMI) of 23.5 to 30 kg/m^2^. The subjects received and fully understood the detailed description of this human study, voluntarily decided to participate, and agreed in writing to comply with the precautions.

Subjects who met any of the following criteria were excluded from this study:

1) those who had lost more than 10% of their body weight within three months before the screening test; 2) those who had taken medicines or health supplements related to detoxification or weight loss within one month prior to the screening test; 3) those with clinically significant acute or chronic diseases of the cardio-cerebrovascular system, endocrine system, immune system, respiratory system, hepatobiliary system, kidney and urinary system, nervous system, or musculoskeletal system or with inflammatory diseases or blood tumors; 4) those with a history of gastrointestinal disease (e.g., Crohn’s disease) or gastrointestinal surgery (excluding simple appendectomy or herniotomy) that could affect absorption of the study diet; 5) those with hypersensitivity reactions to the ingredients of the study diet; 6) those who had taken antipsychotic drugs or narcotic analgesics within six months prior to the screening test; 7) those suspected of drug abuse or a history thereof; 8) those drinking alcohol in excess of 21 units/weeks or have a history of alcohol abuse; 9) those with serum AST or ALT level three times greater than the upper limit of the reference range, or serum creatinine level over 2.0 mg/dL in diagnostic examinations; 10) those who had participated in other studies within two months of the screening test; 11) those who were in menopause (for more than 12 months) or perimenopause period (for a continuous period of 3 months or more); 12) those who were pregnant, breastfeeding, or planning to become pregnant during the study; 13) those who did not agree with the use of effective contraception methods (condoms, contraceptives, intrauterine contraceptives, and male partners with vasectomy) during the study period; and 14) those who were deemed unfit for this study by the tester due to diagnostic examination results or other reasons.

### Study design

Among 61 volunteers who provided written consent to participate in this study, 45 who met the selection and exclusion criteria were selected. Among the 45 selected subjects, 30 were assigned 1:1 to the test group and control group 1 by a single-blind and random arrangement method, and 15 were assigned to control group 2. A total of 45 participants were randomly assigned into one of the study groups (15 subjects each) using a computer-generated random number table by the Randomization program of the version 9.2 SAS® system (SAS Institute, Cary, NC, USA). The subjects were to consume the respective study diets for four weeks (test group, WD; control group 1, CRD; control group 2, MRD). The average daily energy values of the menus provided to the test group and control group 1 are shown in Table 1. All 45 of the registered subjects completed all the procedures and examinations specified in the test plan.

**Table 1:** Provided mean dietary contents of the wellness diet group (per day)

Diets	WD (Wellness-up diet)					CRD(Calorie-restricted diet)				
	Breakfast	Lunch	Dinner	Snack	Total	Breakfast	Lunch	Dinner	Snack	Total
	Nutri shake (6 kinds of whole food in a powder)	Mixed fruit and vegetable juice	Salad (organic ingredients)	Fruit and nut-based food bars		Shake (commercial yogurt powder)	Fruit and vegetable juice (commercial)	Salad (common ingredients)	Fruit and nut-based food bars	
**Weight (g)**	58	450	400	60		50	450	400	60	
**Energy (kcal)**	274.8	246.2	509.4	194.3	1225	245.0	266.0	509.4	194.3	1215
**CHO (g)**	49.5	70.2	35	13.9	168.6 (55%)	46.0	60.5	35	13.9	155.4 (51%)
**Protein (g)**	6.4	4.6	22.7	5.7	39.4 (12%)	7.0	2.7	22.7	5.7	38.1 (13%)
**Fat (g)**	3.2	0.6	31	12.9	47.7 (33%)	3.6	1.0	31	12.9	48.5 (36%)
**Fiber (g)**	3	13	7.1	3.7	26.8	–	9.0	7.1	3.7	19.8

*Abbreviations*: *WD* Wellnessup diet, *CRD* calorie-restricted diet

### Dietary interventions

#### Experimental diet (Wellnessup diet: WD)

The diet of the test group (WD) consisted of organic ingredients produced by smart farms (pesticide-free and pollution-free). The menu consisted of breakfast (shakes), lunch (fresh fruit and vegetable juice), dinner (salads), and snacks (nut bars) and was followed on 14 days cycle. The ingredients in the shakes included whole-food materials to promote detoxification while supplementing grain, and fruits powder. The shakes were delivered in powder form in individually packed sticks, which were to be opened, poured into a shake bottle, combined with at least 250 mL of water (the amount of water used is a matter of personal preference), and mixed well before consumption. For lunch, 450 mL of vegetable juice (a form made by grinding various raw fruits and vegetables) was refrigerated and shaken before consumption. For dinner, the salad consisted of about 400 g of organic green vegetables, whole grains, meat, and fruit, along with a salad dressing. The nut bars was made using a variety of dried fruits and roasted nuts. Which was recommended as snacks between lunch and dinner and were to be stored at room temperature (Supplementary Table 1).

#### Control group 1 (calorie-restricted diet: CRD)

The diet of control group 1 was similar to the WD in its dietary weight and caloric content. This calorie-restricted diet consisted of a shake for breakfast, fruit and vegetable juice for lunch, salad for dinner, and nut bars for snacks, as in the test group. The CRD was composed of general ingredients purchased from supermarkets. Shake powder products for market sale (grain and yogurt) were used for breakfast, and fruit juice products for market sale were served for lunch. Salads consisting of vegetables, fruit, meat, and whole grains cultivated by general farming were served for dinner, and nut bars were provided as snacks. For breakfast, the shake was prepared through addition of at least 250 mL of water (based on personal preference) in a shake bottle, mixed well before consumption. For lunch, 450 mL of refrigerated vegetable juice was shaken and ingested. For dinner, the salad consisted of about 400 g of green vegetables, whole grains, meat, and fruit, along with a salad dressing. The nut bars were recommended as snacks between lunch and dinner and were to be stored at room temperature.

#### Control group 2 (maintaining regular diet: MRD)

The subjects assigned to control group 2 were required to maintain their daily meal patterns without calorie restriction for four weeks and did not receive provided test meals. The subjects recorded all foods and beverages consumed for 28 days in detail on a diet record form.

#### Compliance and safety of the study subjects

We recommended that the subjects eat only the clinical research diet provided during the study period and asked them to maintain the same level of physical activity that they were performing before the study to minimize the impact of lifestyle changes on the test results. To observe changes in diet and physical activity, we assessed the dietary intake, diet compliance, and physical activity levels of the subjects during the study period. For examination of diet, subjects were asked to record the foods they consumed every day in a dietary diary. Subjects were monitored for current drug use, self-reported symptoms or side effects, changes in physical activity, lifestyle habits, and suitability for the chosen diet. A safety test was conducted to evaluate the clinical condition of each subject, including adverse reactions, and the results were recorded in a case report to investigate possible adverse reactions in this human study. In addition, vital signs, blood tests, and chemical tests were reviewed.

### Outcome measures

#### Anthropometric measures, harmful elements in hair, and biochemical analysis

All subjects underwent efficacy and safety assessments before and after the four-week study. The primary efficacy evaluation items were harmful elements in hair (29 types of heavy metals), and the secondary efficacy evaluation items were anthropometric indexes (weight, BMI, body fat, body fat percentage, fat-free mass, WC, HC, and waist-to-hip ratio(WHR)), lipid metabolic indexes (total cholesterol, Apo A1, Apo B, triglycerides, HDL-cholesterol, and LDL-cholesterol levels), blood glucose indexes (glucose, insulin, and HbA1c), an inflammatory index (hs-CRP), uric acid level, GGT level, and urinary organic acid levels (β-hydroxybutyrate, isocitrate, methylmalonate, α-ketoisocaproate, α-hydroxybutyrate, 3,4-dihydroxyphenylpropionate, and 8-hydroxy-2-deoxyguanosine). As safety measurements, abnormal responses were monitored, a diagnostic examination was performed, vital signs were tested, a physical examination was conducted, and an electrocardiogram was recorded.

### Anthropometry and medical examination

The anthropometric measures were height, weight, WC, HC, and BMI. Height and weight were measured with a GL-150 (G-Tech Co., Uijeongbu, Korea) while subjects were dressed in light clothing. WC was measured with a tape measure around the middle of the pelvis and the lower part of the ribs while the subject stood with his/her feet 25–30 cm apart and breathed comfortably. Body fat, body fat percentage, and muscle mass were measured with an Inbody 720 (*Biospace* Co., Seoul, Korea).

#### Blood pressure measurement

Blood pressure was measured with an HBP-9020 (Omron Healthcare Co., Ltd., Kyoto, Japan) after the subject had arrived at the research site and had rested comfortably for at least 10 min. Three measurements of systolic blood pressure (SBP), diastolic blood pressure (DBP), and pulse rate were recorded at intervals of approximately 2 min while the subject was seated, and the average was calculated. The medical team carried out the examination through interviews, ocular inspection, auscultation, percussion, and palpation.

#### Harmful elements in hair (29 types of heavy metals)

In total, 29 harmful elements were measured in hair by means of an ICP, Agilent 7800 ICP-MS (Agilent, CA, USA) [35]. Twelve carcinogenic heavy metals (arsenic, beryllium, cadmium, lead, mercury, nickel, palladium, rhodium, thallium, tin, thorium, and uranium) and 17 toxic heavy metals (aluminum, antimony, barium, bismuth, cerium, cesium, gadolinium, gallium, gold, indium, platinum, rubidium, silver, tellurium, titanium, tungsten, and zirconium) were identified in hair. For collection, 0.4 g of hair was cut as close to the roots as possible from 5 to 6 places in the occipital region. Hair was also collected at the nearest point to the scalp (two to three cm from the scalp), and long hairs or long hair ends were not used. It was recommended that the subjects wash their hair with only water on the day of hair sampling (sweaty or dirty hair was not suitable).

#### Blood test

Blood was collected after subjects had fasted for more than 12 h overnight. The blood was centrifuged at 3000 rpm (Hanil Science Industrial Co., Ltd. Seoul, Korea) for 20 min and kept frozen at − 80 °C until analysis. Total cholesterol, neutral blood lipid, and HDL-C levels were analyzed with a Hitachi 7600–100 analyzer (Hitachi High Technologies Corporation, Tokyo, Japan), and the LDL-C content was calculated with the Friedewald formula [36]. Glucose, insulin and HbA1c levels were measured to investigate blood glucose control. Lipid metabolic indexes of free fatty acid, apolipoprotein A1, apolipoprotein B, and apolipoprotein E, along with liver enzyme indexes of GGT, ALT, AST and total bilirubin, were analyzed with an ADVIA 2400 chemistry system (SIEMENS, Munich, Germany).

#### Urinary organic acid test

The intermediate urine of the first urine of the morning (about 10 mL) was collected from each subject, and urinary organic acid tests were conducted. The urine samples were analyzed for levels of β-hydroxybutyrate, isocitrate, α-ketoisocaproate, methylmalonate, and α-hydroxybutyrate (AHB) by GC-MS (Agilent 5977B Series GCC/MSD System, Santa Clara, CA, USA). The 8-hydroxy-2-deoxyguanosine (8-OHdG) level was analyzed by LC-MS (Agilent 6400 Series Triple Quad LC/MS System). The water intake of the subjects was limited to one cup (240 mL) after 8 p.m. the day before urine sampling. The subjects were also instructed to refrain from excessive concentrate, drinks, coffee, and alcohol.

#### Investigation of dietary intake and physical activity

Trained nutritionists explained the dietary diary to each subject and provided instructions for use. The dietary intake surveys were collected at the first (baseline) and second visits, and the results were verified directly through interviews when the data were retrieved. The subjects completed the first dietary intake survey three days prior to the first visit in the dietary diary according to the meal record method. The purpose of the first survey was to determine the total calorie and nutrient composition of the average daily diet before the study. The second dietary intake survey was conducted to determine the intake of the study diet and all the other foods (beverages) for 28 days. The subjects assigned to the MRD group recorded all the foods they ate freely every day in the diary. The randomly assigned subjects in the WD and CRD groups marked whether or not they had eaten the study diet each day in the issued diary. The subjects in these groups also recorded their intake levels of the provided foods and photographed the remaining amounts after they had eaten the meals each day. Other foods and beverages consumed were also recorded in detail on a separate form. Even if the subjects ate meals other than those provided in this study, they were not eliminated. The subjects were taught to accurately record the additional foods that they would inevitably consume, after receiving training to eat only the foods that were provided and those that were added to the served foods. In the analysis of dietary intake data, the average values of the dietary diaries for 28 days were evaluated by the researchers assigned to each group in the Can Pro 4.0 program (The Korean Nutrition Society, Seoul, Korea). Physical activity was evaluated according to a metabolic equivalent task (MET) assessment using the global physical activity questionnaire (GPAQ). The MET value was used for analysis of physical activity or GPAQ data, representing the relative proportion of working metabolic rate to metabolic rate at rest.

### Statistical analysis

All statistical analyses were performed using SAS® version 9.2 (SAS Institute, Cary, North Carolina, USA). The data was presented as mean ± standard deviation (SD) or median with interquartile range (25th to 75th percentile), depending upon normalcy of data distribution.

The Chi-square test and Wilcoxon rank-sum test were used for the homogeneity test and the baseline homogeneity test, respectively, among the study groups. The variation in primary and secondary outcomes efficacy evaluation item after four weeks of diet intake (Δ Week 4 - Week 0) was compared among the WD, CRD, and MRD groups by the Kruskal-Wallis test and the Repeated Measures ANOVA. Control groups 1 and 2 were compared with the test group by the Wilcoxon rank-sum test. For each group (WD, CRD, and MRD group), the changes in intake before and after the four-week study were evaluated by the Wilcoxon signed-rank test. The significance was statistically significant at the level of *p* < 0.05.

### Sample size

The sample size was statistically calculation for this study was based on the assumption of − 2.6 kg variation in measured weight after 7 days of Lemon detox diet supplementation, − 0.6 kg variation in the control group, and standard deviation of 1.7 kg, a sample size of each group was determined to be 15 participants, allowing for a 20% dropout rate. The groups were equal in size in order to obtain the greatest statistical power. The number of subjects required was calculated as described in Kim et al. (2015) [16] therefore, a total of 45 participants were randomly assigned into one of the study groups.

## Results

### Participant demographic characteristics

**Fig. 1:**
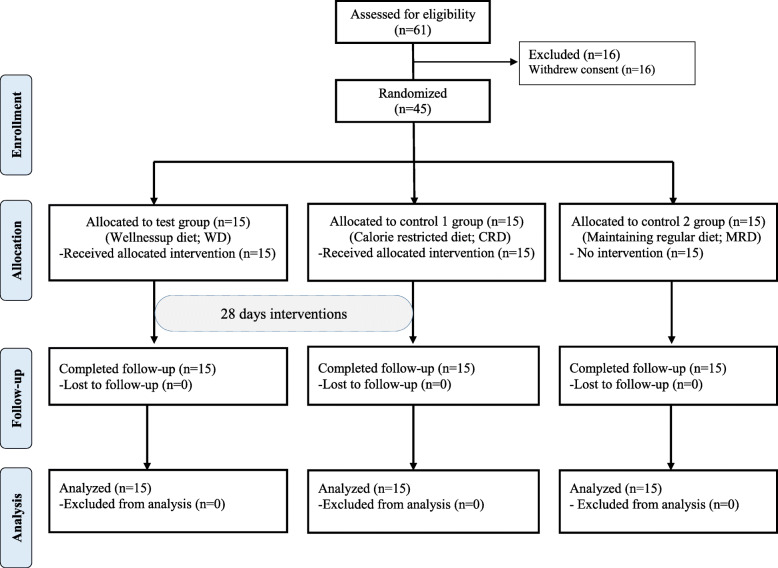
Flow diagram of the participants in this study

The general characteristics of the subjects in this study are presented in Table 2**.** All the study subjects were women, and the average age was 26.2 ± 4.6 years. The age, weight, BMI, blood pressure, pulse, blood glucose and lipid profiles, drinking history, and smoking history did not differ significantly among the three groups. Sixty-one subjects voluntarily provided written consent and were screened for this study, and 45 of them were selected following evaluation of suitability for the study. No one was allowed to drop out or violate the research plan during this study, and the 45 registered subjects completed all the study procedures **(**Fig. 1**).**

**Table 2:** General characteristics of the subjects

Variables	WD (***n*** = 15)	CRD (***n*** = 15)	MRD (***n*** = 15)	***p***-value^**1)**^ (W-M-C)	***p***-value^**2)**^(W-M)	***p***-value^**3)**^(W-C)	***p***-value^**4)**^(M-C)
Age, years	25.73 ± 4.18	24.93 ± 4.57	27.93 ± 5.04	0.261	0.602	0.234	0.138
Sex (male/female)	0/15	0/15	0/15	–	–	–	–
Height (cm)	163.33 ± 5.47	161.80 ± 5.05	162.93 ± 6.65	0.683	0.347	> 0.99	0.617
Weight (kg)	70.21 ± 8.41	67.84 ± 6.29	70.18 ± 6.59	0.687	0.534	> 0.99	0.407
Body Mass Index (kg/m^2^)	26.25 ± 1.98	25.91 ± 1.95	26.41 ± 1.60	0.455	0.507	0.470	0.280
Body Fat Mass (kg)	25.05 ± 4.67	23.69 ± 4.48	25.18 ± 4.69	0.547	0.361	0.803	0.361
Body Fat (%)	35.89 ± 3.42	35.25 ± 4.00	36.04 ± 4.36	0.586	0.455	0.724	0.361
Fat-Free Mass (kg)	44.54 ± 5.51	43.13 ± 3.70	44.33 ± 4.03	0.713	0.756	0.819	0.361
Waist Circumference (cm)	88.46 ± 7.84	85.67 ± 7.53	87.51 ± 6.14	0.633	0.372	0.884	0.493
Hip Circumference (cm)	101.49 ± 3.50	99.75 ± 3.48	101.87 ± 4.66	0.334	0.254	0.884	0.178
Drinking (yes/no)^5)^	8/7	6/9	7/8	0.765^3)^	0.464	0.715	0.713
Alcohol (units/week)	2.51 ± 0.53	2.55 ± 2.62	4.70 ± 3.19	0.287	0.212	0.415	0.247
Current Smoker (yes/no)	0/15	0/15	0/15	–	–	–	–
TC (mg/dL)	196.93 ± 32.84	182.13 ± 29.34	207.27 ± 38.31	0.213	0.198	0.561	0.115
TG (mg/dL)	96.13 ± 44.13	74.07 ± 27.14	107.20 ± 64.91	0.215	0.171	0.709	0.120
HDL-C (mg/dL)	63.73 ± 14.10	63.47 ± 13.23	67.87 ± 17.08	0.612	0.950	0.394	0.418
LDL-C (mg/dL)	108.27 ± 27.83	96.93 ± 26.42	113.40 ± 33.87	0.324	0.281	0.868	0.152
Glucose (mg/dL)	92.20 ± 11.19	85.00 ± 5.20	86.07 ± 7.41	0.083	0.038	0.092	0.819
Insulin (μU/mL)	11.80 ± 4.49	9.29 ± 4.13	12.14 ± 8.46	0.230	0.115	0.299	0.407
HbA1c (%)	5.41 ± 0.21	5.29 ± 0.21	5.37 ± 0.36	0.430	0.177	0.564	0.596
hs-CRP (mg/L)	1.80 ± 2.99	0.96 ± 1.87	0.95 ± 1.48	0.317	0.183	0.443	0.319
GGT (IU/L)	27.87 ± 48.53	10.00 ± 4.21	14.53 ± 7.49	0.184	0.191	0.983	0.069
Uric acid (mg/dL)	5.21 ± 1.08	4.74 ± 1.06	5.13 ± 0.82	0.264	0.152	0.934	0.184

Values are presented as mean ± SD

^1)^ Analyzed by the Kruskal-Wallis test for change in WD-MRD-CRD groups

^2)^ Analyzed by the Wilcoxon rank-sum test for change in WD-MRD groups

^3)^ Analyzed by the Wilcoxon rank-sum test for change in WD-CRD groups

^4)^ Analyzed by the Wilcoxon rank-sum test for change in MRD-CRD groups

^5)^ Analyzed by the Chi-square test

*Abbreviations*: *WD* Wellnessup diet, *MRD* maintaining regular diet, *CRD* calorie-restricted diet, *TC* Total Cholesterol, *TG* Triglycerides, *HDL-C* HDL-Cholesterol, *LDL-C* LDL-Cholesterol, *GGT* Gamma-Glutamyl Transferase

### Changes in harmful elements in hair

The changes in toxic trace elements in hair are presented in Table 3. A total of 29 heavy metals, three were significantly lower or exhibited a reduction after four weeks of WD intake compared to baseline (Ni, *p* = 0.003; Rh, *p* = 0.005; W, *p* = 0.002). Among these four heavy metals were significantly lower in the WD group compared to the CRD or MRD group (Ni, *p* = 0.025; Rh, *p* = 0.034; Sn, *p* = 0.050; Ga, *p* = 0.042).

**Table 3:** Changes in concentrations (μg g^−1^) of harmful elements in hair

Variables	WD (***n*** = 15)	CRD (***n*** = 15)	MRD (***n*** = 15)	***p***-value^**2)**^ (W-M-C)	***p***-value^**3)**^ (W-M)	***p***-value^**4)**^ (W-C)	***p***-value^**5)**^ (M-C)
**Pb**							
Baseline	4.6(3.2–7.6)	5.7(3.4–9.3)	4.3(3.4–6.6)				
4-week	3.3(2.6–4.9)	5.6(3.1–9.6)	3.6(2.9–6.8)	0.668	0.443	0.819	0.547
*p*-value^1)^	0.080	0.454	0.178				
**Ni**							
Baseline	0.9(0.7–1.4)	1.0(0.3–1.3)	0.9(0.5–1.2)				
4-week	0.6(0.4–1.0)	0.6(0.5–1.3)	1.0(0.6–1.5)	0.063	0.228	**0.025**	0.212
*p*-value	**0.003**	0.254	0.600				
**Rh**							
Baseline	0.0004(0.0002–0.0006)	0.0004(0.0003–0.0007)	0.0004(0.0002–0.0004)				
4-week	0.0002(0.0–0.0004)	0.0004(0.0002–0.0007)	0.0003(0.0002–0.0004)	0.067	**0.034**	0.070	0.770
*p*-value	**0.005**	0.748	0.578				
**Be**							
Baseline	0.0037(0.0032–0.0058)	0.0042(0.0024–0.0068)	0.0041(0.0020–0.0062)				
4-week	0.0029(0.0025–0.0038)	0.0034(0.0024–0.0096)	0.0055(0.0019–0.0099)	0.292	0.431	0.097	0.648
*p*-value	0.147	0.627	0.235				
**As**							
Baseline	0.22(0.15–0.37)	0.23(0.15–0.31)	0.18(0.14–0.29)				
4-week	0.20(0.15–0.28)	0.19(0.15–0.25)	0.20(0.13–0.28)	0.534	0.480	0.468	0.361
*p*-value	0.262	**0.041**	0.989				
**Hg**							
Baseline	7.17(5.21–11.63)	6.56(4.36–8.91)	9.06(6.91–14.99)				
4-week	8.37(5.95–10.51)	6.89(4.52–9.71)	10.05(7.38–15.72)	0.585	0.934	0.455	0.320
*p*-value	0.639	0.229	0.107				
**U**							
Baseline	0.22(0.07–0.37)	0.18(0.03–0.61)	0.14(0.01–1.52)				
4-week	0.12(0.04–0.35)	0.18(0.03–0.43)	0.10(0.02–1.81)	0.596	0.329	0.520	0.771
*p*-value	0.098	0.734	0.798				
**Sn**							
Baseline	0.61(0.29–2.35)	0.38(0.30–0.77)	0.53(0.40–1.21)				
4-week	0.55(0.35–0.95)	0.44(0.28–1.07)	0.59(0.33–1.98)	0.090	**0.050**	0.068	0.852
*p*-value	0.073	0.561	0.421				
**Cd**							
Baseline	0.03(0.02–0.06)	0.03(0.02–0.04)	0.03(0.02–0.05)				
4-week	0.04(0.03–0.06)	0.03(0.02–0.05)	0.03(0.02–0.05)	0. 774	> 0.999	0.551	0.550
*p*-value	0.482	0.394	0.871				
**Tl**							
Baseline	0.0024(0.0014–0.0052)	0.0045(0.0031–0.0070)	0.0025(0.0014–0.0045)				
4-week	0.0024(0.0020–0.0059)	0.0053(0.0020–0.0065)	0.0041(0.0020–0.0051)	**0.032**	0.221	0.206	**0.008**
*p*-value	0.610	0.223	0.031				
**Th**							
Baseline	0.0034(0.0022–0.0046)	0.0026(0.0014–0.0038)	0.0026(0.0020–0.0034)				
4-week	0.0032(0.0023–0.0039)	0.0030(0.0016–0.0040)	0.0025(0.0019–0.0031)	0.589	0.254	0.709	0.740
*p*-value	0.945	0.323	0.820				
**Pd**							
Baseline	0.013(0.008–0.024)	0.009(0.007–0.017)	0.024(0.006–0.033)				
4-week	0.014(0.007–0.016)	0.011(0.007–0.017)	0.016(0.011–0.032)	0.583	0.290	0.547	0.819
*p*-value	0.104	> 0.999	0.703				
**Gd**							
Baseline	0.002(0.002–0.003)	0.002(0.001–0.002)	0.002(0.002–0.003)				
4-week	0.002(0.001–0.003)	0.002(0.001–0.003)	0.002(0.001–0.004)	0.534	0.362	0.330	> 0.999
*p*-value	0.139	> 0.999	0.945				
**Ga**							
Baseline	0.016(0.010–0.021)	0.012(0.006–0.019)	0.012(0.009–0.016)				
4-week	0.010(0.008–0.016)	0.010(0.065–0.014)	0.013(0.011–0.027)	**0.042**	0.213	**0.014**	0.198
*p*-value	0.064	0.804	0.135				
**Au**							
Baseline	0.0(0.0–0.1)	0.1(0.0–0.3)	0.1(0.0–0.2)				
4-week	0.1(0.0–0.2)	0.1(0.0–0.1)	0.1(0.0–0.3)	0.669	0.490	0.907	0.438
*p*-value	0.313	0.883	0.234				
**Rb**							
Baseline	0.229(0.152–1.219)	0.430(0.187–0.662)	0.294(0.155–0.422)				
4-week	0.350(0.145–0.978)	0.126(0.159–0.563)	0.161(0.133–0.276)	0.825	0.619	0.619	0.836
*p*-value	0.847	0.277	0.229				
**Ba**							
Baseline	11.03(4.55–29.45)	8.25(5.29–10.76)	20.61(9.82–32.33)				
4-week	9.74(5.51–21.31)	8.67(6.35–15.88)	16.45(12.56–24.63)	0.589	0.740	0.534	0.320
*p*-value	0.847	0.934	0.208				
**Pt**							
Baseline	0.001(0.0–0.001)	0.001(0.0–0.001)	0.001(0.0–0.01)				
4-week	0.0(0.0–0.001)	0.0(0.0–0.001)	0.0(0.0–0.001)	0.777	0.509	0.891	0.616
*p*-value	0.531	> 0.999	> 0.999				
**Bi**							
Baseline	0.027(0.012–0.131)	0.014(0.008–0.045)	0.020(0.007–0.108)				
4-week	0.038(0.020–0.050)	0.023(0.011–0.036)	0.022(0.007–0.054)	0.690	0.384	0.575	> 0.999
*p*-value	0.248	0.891	> 0.999				
**Ce**							
Baseline	0.025(0.016–0.074)	0.021(0.013–0.033)	0.017(0.010–0.022)				
4-week	0.028(0.020–0.081)	0.020(0.013–0.036)	0.017(0.012–0.050)	0.899	0.772	0.648	> 0.999
*p*-value	0.463	0.534	0.552				
**Cs**							
Baseline	0.0(0.0–0.01)	0.0(0.0–0.01)	0.0(0.0–0.0)				
4-week	0.0(0.0–0.01)	0.0(0.0–0.0)	0.0(0.0–0.0)	0.213	0.173	0.976	0.115
*p*-value	0.625	0.625	0.500				
**Sb**							
Baseline	0.083(0.063–0.162)	0.067(0.059–0.086)	0.069(0.042–0.087)				
4-week	0.087(0.066–0.164)	0.083(0.069–0.089)	0.073(0.050–0.106)	0.900	0.576	0.934	0.934
*p*-value	0.352	0.208	0.352				
**Al**							
Baseline	26.2(18.9–52.8)	33.2(26.9–36.7)	27.6(23.3–39.3)				
4-week	29.4(19.4–55.3)	37.1(28.8–44.5)	28.3(19.7–50.4)	0.809	0.648	0.534	> 0.999
*p*-value	0.934	0.463	0.367				
**Ag**							
Baseline	0.30(0.22–1.18)	0.85(0.24–1.67)	0.30(0.14–0.90)				
4-week	0.24(0.18–1.42)	0.94(0.28–1.82)	0.32(0.15–0.88)	0.218	0.191	0.097	0.836
*p*-value	0.092	0.561	0.552				
**In**							
Baseline	0.0(0.0–0.0)	0.0(0.0–0.0)	0.0(0.0–0.0)				
4-week	0.0(0.0–0.0)	0.0(0.0–0.0)	0.0(0.0–0.0)	0.398	> 0.999	0.351	0.351
*p*-value	–	–	–				
**Zr**							
Baseline	0.14(0.10–0.19)	0.13(0.08–0.24)	0.10(0.08–0.15)				
4-week	0.10(0.06–0.20)	0.10(0.08–0.19)	0.08(0.06–0.11)	0.670	0.560	0.724	0.418
*p*-value	0.382	0.717	0.133				
**W**							
Baseline	0.025(0.013–0.030)	0.025(0.010–0.046)	0.021(0.012–0.036)				
4-week	0.014(0.008–0.019)	0.008(0.007–0.014)	0.015(0.010–0.021)	0.217	0.633	0.158	0.135
*p*-value	**0.002**	**0.015**	0.337				
**Te**							
Baseline	0.002(0.001–0.005)	0.003(0.002–0.005)	0.0(0.0–0.001)				
4-week	0.002(0.0–0.003)	0.002(0.0–0.005)	0.001(0.0–0.002)	0.270	0.754	0.134	0.228
*p*-value	0.173	0.315	0.652				
**Ti**							
Baseline	18.2(9.0–63.1)	17.7(10.5–55.2)	18.6(13.7–42.0)				
4-week	19.3(8.8–56.0)	20.9(11.7–29.5)	19.9(13.6–30.8)	0.999	> 0.999	0.901	0.984
*p*-value	0.793	0.858	0.571				

Values are presented as median (interquartile range)

^1)^ Analyzed by the Wilcoxon signed-rank test

^2)^ Analyzed by the Kruskal Wallis test for WD-CRD-MRD groups

^3)^ Analyzed by the Wilcoxon rank-sum test for change in WD-MRD groups

^4)^ Analyzed by the Wilcoxon rank-sum test for change in WD-CRD groups

^5)^ Analyzed by the Wilcoxon rank-sum test for change in MRD-CRD groups

*Abbreviations*: *WD* Wellnessup diet, *MRD* maintaining regular diet, *CRD* calorie-restricted diet

### Changes in anthropometric indexes

The results of the anthropometric indexes are presented in Table 4. After four weeks of diet intake, the WD group exhibited significant decreases in weight (*p* = 0.013), BMI (*p* = 0.011), fat-free mass (*p* = 0.002), and WC (*p* = 0.0067) compared to baseline. In the WD group compared to the CRD or MRD group weight (*p* = 0.007), body mass index (*p* = 0.001), and WC (*p* = 0.007) were significantly lower after four weeks of participation in the diet. Reduction change of fat-free mass was also significantly lower in the WD group than in the CRD or MRD group (*p* = 0.002). Although fat-free mass was reduced in both the WD group (*p* = 0.046) and the CRD group (*p* = 0.019), a greater decrease was observed in the CRD group.

**Table 4:** Changes in the anthropometric parameters of the subjects measured at baseline and 4-week of the study

Variables	WD (***n*** = 15)	CRD (***n*** = 15)	MRD (***n*** = 15)	***p***-value^**2)**^(W-M-C)	***p***-value^**3)**^(W-M)	***p***-value^**4)**^(W-C)	***p***-value^**5)**^(M-C)
**Weight (kg)**							
Baseline	69.59 ± 8.98	66.82 ± 6.59	69.51 ± 6.97				
4-week	67.71 ± 9.93	63.60 ± 6.11	69.29 ± 6.98	**0.001**	0.125	**0.050**	**0.001**
*p*-value^1)^	**0.013**	**0.001**	0.322				
**Body Mass Index (kg/m**^**2**^**)**							
Baseline	26.02 ± 2.27	25.50 ± 2.12	26.15 ± 1.83				
4-week	25.30 ± 2.72	24.27 ± 1.99	26.07 ± 1.89	**0.004**	0.164	**0.046**	**0.001**
*p*-value	**0.011**	**0.001**	0.296				
**Body Fat Mass (kg)**							
Baseline	25.05 ± 4.67	23.69 ± 4.48	25.18 ± 4.69				
4-week	24.26 ± 5.23	22.55 ± 4.19	25.13 ± 4.80	0.115	0.534	0.262	**0.049**
*p*-value	0.131	**0.014**	0.719				
**Fat Free Mass (kg)**							
Baseline	44.54 ± 5.51	43.13 ± 3.70	44.33 ± 4.03				
4-week	43.45 ± 5.97	41.05 ± 3.55	44.16 ± 3.86	**0.001**	**0.046**	**0.019**	**0.001**
*p*-value	**0.002**	**0.001**	0.366				
**Waist Circumference (cm)**							
Baseline	88.46 ± 7.84	85.67 ± 7.53	87.51 ± 6.14				
4-week	85.80 ± 8.81	81.40 ± 6.35	86.64 ± 6.13	**0.009**	0.184	0.135	**0.008**
*p*-value	**0.007**	**0.001**	0.367				
**Hip Circumference (cm)**							
Baseline	101.49 ± 3.50	99.75 ± 3.48	101.87 ± 4.66				
4-week	99.87 ± 4.36	97.35 ± 3.93	101.64 ± 5.05	**0.006**	0.361	**0.040**	**0.002**
*p*-value	**0.010**	**0.001**	0.366				
**Waist to Hip Ratio**							
Baseline	0.87 ± 0.05	0.86 ± 0.07	0.86 ± 0.05				
4-week	0.86 ± 0.06	0.84 ± 0.06	0.85 ± 0.05	0.233	0.393	0.303	0.138
*p*-value	**0.050**	**0.009**	0.473				

Values are presented as mean ± SD or number

^1)^ Analyzed by the Wilcoxon signed-rank test

^2)^ Analyzed by the Repeated Measures ANOVA according to the satisfaction of normality

^3)^ Analyzed by the Wilcoxon rank-sum test for change in WD-MRD groups

^4)^ Analyzed by the Wilcoxon rank-sum test for change in WD-CRD groups

^5)^ Analyzed by the Wilcoxon rank-sum test for change in MRD-CRD groups

*Abbreviations*: *WD* Wellnessup diet, *MRD* maintaining regular diet, *CRD* calorie-restricted diet

### Changes in urinary organic acids

The results for the urinary organic acids are presented in (Supplementary Table 2).

The levels of isocitrate (*p* = 0.041) and α-ketoisocaproate (*p <* 0.001) in the WD group were significantly increased after the diet than at baseline. In the CRD group, the levels of β-hydroxybutyrate (*p* = 0.003), methylmalonate (*p* = 0.002), and 8-hydroxy-2-deoxyguanosine (*p* = 0.030) were significantly different after four weeks of the diet. Significant changes in methylmalonate (*p* = 0.041) and 8-hydroxy-2-deoxyguanosine levels (*p* < 0.001) were observed in the MRD group after four weeks. The change in methylmalonate level differed significantly between the WD and MRD groups (*p* = 0.028).

### Changes in average daily diet intake and physical activity

The dietary intake results are presented in Table 5. In terms of the composition of major nutrients and the number of calories consumed, the WD and CRD groups exhibited good dietary compliance, with no significant difference between the groups. The dietary intakes were significantly lower in the WD and CRD groups than in the MRD group (calories, *p* = 0.002; CHO, *p* = 0.001; protein, *p <* 0.0001). The daily intake of dietary fiber in the MRD group was 12.6 g (IQR¼ 9.8 g to 14.6 g), which was significantly lower than that of the WD group 23.0 g (IQR¼ 20.4 g to 23.3 g, *p* < 0.001) or the CRD group 15.6 g (IQR¼ 14.7 g to 16.9 g, *p* < 0.0001). The daily intake of cholesterol was significantly lower in the WD group than in the CRD and MRD groups (*p* < 0.001). The intakes of antioxidant vitamins such as vitamin A (*p* < 0.001), vitamin E (*p* = 0.004), beta-carotene (*p* < 0.0001), and vitamin C (*p* < 0.0001) were significantly higher in the WD group than in the CRD and MRD groups. The daily intake of folic acid was significantly higher in the CRD group than in the WD and MRD groups (*p* = 0.007), while the daily intakes of vitamins B_1_ (*p* = 0.002) and B_2_ (*p* = 0.002) were significantly higher in the MRD group than in the WD and CRD groups. Physical activity surveys revealed that the body activity (MET) of the CRD and MRD groups tended to decrease during the study that there was no significant difference among the groups(*p* > 0.05).

**Table 5:** Nutrient intakes of the study subjects during the 4-week intervention period

Variables	WD (***n*** = 15)	CRD (***n*** = 15)	MRD (***n*** = 15)	***P***-value^**1)**^ (W-M-C)	***P***-value^**2)**^ (W-M)	***P***-value^**3)**^ (W-C)	***P***-value^**4)**^ (M-C)
**Energy (kcal)**							
Baseline	1443.1(1219.1–1682.4)	1905.2(1435.1–2204.2)	1714.3(1562.2–1942.7)				
4-week	1222.9(1164.9–1265.8)	1135.6(1068.7–1332.2)	1502.5(1335.2–1692.7)	**0.002**	0.481	**0.002**	**0.003**
**Carbohydrate (g)**							
Baseline	215.6(178.2–262.3)	243.6(195.9–297.4)	248.0(177.5–277.6)				
4-week	163.1(154.9–172.1)	141.5(132.7–164.1)	191.0(186.6–230.4)	**0.001**	0.213	**0.003**	**0.001**
**Fat (g)**							
Baseline	39.8(35.5–54.1)	61.9(44.2–80.7)	55.5(48.7–68.6)				
4-week	51.0(42.2–52.5)	50.3(47.4–57.5)	49.2(41.1–59.4)	0.971	0.772	0.648	0.967
**Protein (g)**							
Baseline	55.0(40.0–63.0)	62.6(50.3–91.8)	65.4(54.7–76.8)				
4-week	50.0(39.3–45.8)	42.8(41.1–54.3)	58.5(52.5–66.2)	**< 0.0001**	0.075	**< 0.0001**	**0.004**
**Cholesterol (mg**)							
Baseline	208.1(172.2–273.6)	379.7(236.4–481.7)	259.5(218.9–372.2)				
4-week	55.4(30.4–82.7)	84.8(49.3–117.0)	251.0(193.8–332.8)	**< 0.0001**	0.110	**< 0.0001**	**< 0.0001**
**Fiber (g)**							
Baseline	11.1(8.8–14.0)	15.7(11.7–18.1)	12.7(11.0–18.7)				
4-week	23.0(20.4–23.3)	15.6(14.7–16.9)	12.6(9.8–14.6)	**< 0.0001**	**< 0.0001**	**< 0.0001**	**0.002**
**Calcium (mg)**							
Baseline	347.0(190.5–557.8)	431.6(342.5–549.8)	340.1(265.5–411.8)				
4-week	425.7(406.2–450.5)	340.9(317.4–354.9)	293.6(270.0–408.3)	**< 0.001**	**0.001**	**0.009**	0.281
**Phosphorous (mg)**							
Baseline	686.4(630.0–955.0)	930.7(743.9–1162.5)	821.2(750.3–1005.9)				
4-week	635.3(563.4–682.7)	640.8(607.1–712.9)	787.4(671.5–861.8)	**0.019**	0.481	**0.008**	**0.047**
**Potassium (mg)**							
Baseline	1598.2(1370.0–1986.0)	2016.5(1497.1–2449.1)	1981.3(1468.2–2327.1)				
4-week	1894.6(1838.8–2022.4)	1843.2(1776.9–2131.4)	1805.5(1491.1–1976.6)	0.197	0.772	0.106	0.159
**Zinc (mg)**							
Baseline	7.22(6.36–8.79)	8.69(7.31–11.4)	8.81(7.47–10.3)				
4-week	5.19(5.03–6.11)	6.08(5.63–7.19)	8.10(7.56–9.27)	**< 0.0001**	0.025	**< 0.0001**	**0.003**
**Vitamin A (μg RE)**							
Baseline	445.2(384.4–701.4)	616.8(371.5–898.6)	491.0(373.7–875.7)				
4-week	742.1(727.5–810.3)	355.9(335.6–412.9)	448.8(389.5–612.8)	**< 0.0001**	**< 0.0001**	**< 0.0001**	**0.034**
**Vitamin E (mg)**							
Baseline	9.34(7.95–10.5)	15.4(12.2–18.9)	12.6(10.7–15.9)				
4-week	13.7(13.0–13.8)	12.1(11.8–13.0)	11.5(7.3–12.8)	**0.004**	**0.038**	**0.002**	0.147
**Vitamin C (mg)**							
Before	40.9(28.1–146.3)	71.7(45.8–121.7)	78.9(42.1–112.9)				
4-week	211.7(200.9–213.8)	203.5(173.1–213.6)	71.9(47.7–80.7)	**< 0.0001**	0.263	**< 0.0001**	**< 0.0001**
**β-carotene (μg)**							
Baseline	1969.8(1385.1–3119.6)	2009.5(1580.4–3595.9)	2228.0(1406.5–4213.2)				
4-week	4319.7(4204.7–4673.4)	1936.0(1809.4–2279.5)	1933.8(1295.9–2697.8)	**< 0.0001**	**< 0.0001**	**< 0.0001**	0.804
**Vitamin B**_**1**_**(mg)**							
Baseline	0.96(0.84–1.08)	1.15(0.93–1.44)	1.18(1.01–1.43)				
4-week	0.83(0.81–0.89)	0.86(0.79–0.92)	0.99(0.93–1.22)	**0.002**	0.619	**0.002**	**0.005**
**Vitamin B**_**2**_**(mg)**							
Baseline	0.88(0.82–1.18)	1.24(0.92–1.52)	1.01(0.84–1.39)				
4-week	0.72(0.70–0.80)	0.64(0.61–0.85)	0.93(0.79–1.09)	**0.002**	0.229	**0.004**	**0.004**
**Vitamin B**_**6**_**(mg)**							
Baseline	1.01(0.92–1.25)	1.56(1.08–1.68)	1.28(1.08–1.65)				
4-week	1.12(1.09–1.27)	1.08(1.00–1.31)	1.17(1.06–1.40)	0.844	0.836	0.678	0.619
**Folic acid (μg)**							
Baseline	291.8(240.5–331.8)	322.9(276.8–461.9)	387.3(204.7–443.5)				
4-week	330.5(327.5–350.5)	387.0(362.7–405.2)	293.8(233.1–366.8)	**0.007**	**0.031**	0.199	**0.004**
**MET value** (min/week)							
Baseline	1520(480–2600)	1440(540–2400)	2000(480–4320)				
4-week	1240(400–3780)	720(360–2400)	580(240–1560)	0.190	0.885	0.125	0.119

Values are presented as median (interquartile range)

^1)^ Analyzed by the Kruskal Wallis test for WD-CRD-MRD groups

^2)^ Analyzed by the Mann-Whitney U test for change between WD-MRD

^3)^ Analyzed by the Mann-Whitney U test for change between WD-CRD

^4)^ Analyzed by the Mann-Whitney U test for change between MRD-CRD

*Abbreviations*: *WD* Wellnessup diet, *MRD* maintaining regular diet, *CRD* calorie-restricted diet, *MET* metabolic equivalent

### Safety of the study subjects

During the period of participation in this study, there were no clinically significant changes in the results of diagnostic examinations, vital signs tests, biochemical markers (hematological test, blood biochemistry test, and urine test), and electrocardiograms among the safety parameters(*p* > 0.05) (Supplementary Table 3).

## Discussion

To the best of our knowledge, no rigorous clinical studies of detox diets have been conducted. However, this study was conducted as a pilot RCT study to assess the detoxification of toxic trace element following four-week intake of the WD and to verify the efficacy and safety of this diet in reducing body fat in overweight young women. Recently, POPs and heavy metals can accumulate in fat and cause health problems, especially abdominal obesity, which is used as an index of metabolic syndrome [37, 38]. Since heavy metals have very long half-lives, so the process of reduction (detoxification) of heavy metals that have s accumulated in the body is known to be very slow process [39]. Nevertheless, we have performed a rigorous trial to determine the weight loss and detoxification effects. Despite the short-term nature of the present study (four weeks), the levels of eight of the 29 measured heavy metals (lead, nickel, rhodium, uranium, tin, gallium, silver, and tungsten) were reduced or displayed a decreasing tendency in the WD group after the diets (*p* < 0.1), and four of these eight items (nickel, rhodium, tin, and gallium) were clearly lower in the WD group than in the control diet groups (CRD and MRD). In contrast, we did not observe any significant reduction in heavy metal levels in the hair of subjects in the MRD group. The decreases in some of the heavy metals in the WD group may have been due to remove of diets to the total body burden of toxicants during the intervention period. Also, it is believed that the mediate that improved action of the phytonutrients and micronutrients included in the WD, the intake of dietary fiber and antioxidant vitamins, and the intake of organic foods produced. According to Cline (2015) [40], reactive oxygen species are also a by-product of phase I liver detoxification activity. After going through the phase I processes liver detoxification, the activated toxicants are often more toxic than their parent compounds. If these activated, intermediate metabolites are not further metabolized via the phase II conjugation liver detoxification pathways. Therefore, to quench the propagation of free-radical activity, adequate protection from antioxidant nutrients is required using a number of plant derivatives. Anthropometric indexes were significantly improved in both the WD and CRD groups compared to the MRD group. Furthermore, we verified that the CRD group lost significantly more weight than the WD and MRD groups, possibly the CRD Group showed significantly decreased due to the subsequent oxidation of fatty acids in subcutaneous tissues due to a decrease in the intake of calorie and CHO. During this study, there was a few similar to calorie intake between the WD and CRD groups but the daily intake of CHO, protein, antioxidant vitamins, and fiber was significantly higher in the WD group than in the CRD group. In general, Individuals who follow a low-CHO diet are known to have develop ketosis. Also, the acetyl CoA produced by incomplete oxidation of fatty acids in the liver produces large amounts of ketone bodies such as β-hydroxybutyrate, acetoacetic acid, and acetone. In fact, the urinary β-hydroxybutyrate level in the CRD group was significantly greater after 4 weeks of the diet. Urinary ketones are detected in premenopausal women complying with a low-carbohydrate and high-protein diet and are associated with serum ketone concentration [41]. This may have been related to abnormalities in glucose metabolism of CRD group. According to Kossoff & Dorward (2008) [42], a reduced blood glucose level due to long-term CHO deficiency is known to cause body protein degradation, similar to what was observed in the CRD group in this study. Significantly, the large decrease in fat-free mass in the CRD group than in the WD group indicates that the weight loss caused by dietary control was accompanied by reduced muscle mass. What is remarkable is that four weeks after the intake of the test food, the decrease in fat free mass in the CRD group was about twice as much as in the WD group. The reason for the large weight loss in the CRD group is that the reduction in calories and CHO intake and lack of protein has resulted in a greater reduction in the fat free mass than in the body fat. In fact, the average daily protein intake of the CRD and WD groups was 43 g and 50 g, respectively, and the CRD group showed a very low level and the WD group was found to be adequate compared to the recommended daily protein intake (Recommended Dietary Allowance, RDA) of 50 g for women aged 19 ~ 29 (Dietary reference intakes for Koreans 2015; KDRIs.) Therefore, it suggests that the CRD group, which represents a significant decrease in the fat free mass since participating in this study, is at greater risk of causing side effects of weight recovery (yo-yo effect) than the WD group. Gary et al. (2013) [43] reported that the low-CHO diet group showed a higher weight loss than the conventional diet group for the first six months in obese adults, but not significant a year later. Thus, it is ideal not only to reduce body fat while maintaining fat-free mass in weight loss, but also strategies to prevent the recurrence of obesity while maintaining a basal metabolic rate and continuous practical measures in long-term daily life will be very necessary.

Similar to our research, a study Yurrita et al. (2017) [26] have reported that significant decreases in body weight, BMI and body fat percentage and greater loss of musculature were observed in the detox diet group(based on juices for 3 days, followed by a hypocaloric diet for 4 days)than in Mediterranean diet group (a hypocaloric Mediterranean diet for 7 days). A study by Lee et al. (2017) [44] indicated that whole grain cereal effectively attenuates obesity-related skeletal muscle atrophy as well as overall obesity in obese mice. Because of supplementation of whole grain cereal, which contains of beneficial minerals, vitamins, and other phytochemicals, it suggests that may have helped not only suppress muscle atrophy but also weight loss by activating the phosphatidylinositol 3-kinase and protein kinase B pathway. Therefore, the different effects of the WD and CRD on body fat, despite the similar compositions of these diets (i.e., the proportion of calories and the large amounts of nutrients), suggest that the fiber, phytochemicals, and micro nutrients included in the WD may have been responsible for the milder reduction of fat-free mass (muscles) in WD group. Analysis of safety indicators following WD and CRD supplementation indicated that any changes were within the normal range and had no clinical significance.

The strengths of the study include the one-phase design, in which all participants started simultaneously. First, we strictly monitored dietary intake by directly providing all meals to our subjects for four weeks and asking them to complete accurate dietary intake investigated. Second, we confirmed objectively that consuming the WD is superior to consuming calorie-restricted diet in detoxification of heavy metals by comparing and evaluating the effects of a healthy meals based on organic plant based diets consumed in daily life rather than considering the intake of only fruits, vegetables, ingredients or beverages (teas, vinegar, lemon juice, salt water, or drinks mixed with micronutrients). Also, a more sophisticated assessment was made by investigating the effectiveness of nutritionally balanced detox diets for weight loss and toxin elimination. However, there are some limitations to this study that should be considered.

First, four-week intake of this diet was found to reduce certain toxic elements (heavy metals) in hair compared to the control group, although there were limitations in determining the superiority of the test or control diet due to the nature of typical tests for verifying statistical hypotheses. Second, the contents of harmful elements of test meals before the study was not investigated for test meals, so it was not possible to explain the causal relationship a before and after the study. Third, an intake period of long term would be required to verify the efficacy of the diet based measure for discharging heavy metals in hair. Fourth, the assessment of detoxification of heavy metals and POPs requires an integrated analysis of urine as well as hair analysis. Fifth, the number of people who participated in this study was somewhat small, which could limit the generalizability of the results. Therefore, we would expect to observe more pronounced detox improvements in a corroborative study with a greater number of subjects and a longer follow-up period.

## Conclusions

In conclusion, our results suggest that the WD detox program might have several beneficial effects and safety such as body fat reduction and improving some the element detoxification through caloric restriction. This study lays the scientific foundation for a subsequent large-scale corroborative clinical study.

## Supplementary information

**Additional file 1.** CONSORT 2010 checklist *.

**Additional file 2: Table S1.** Provided mean dietary menu of test meals (per day) samples. **Table S2.** Changes in organic acid on urine. **Table S3.** Laboratory profiles of the participants in this study.

## Data Availability

The datasets generated and/or analyzed during the current study are not publicly available to protect patient confidentiality but are available from the corresponding author on reasonable request.

## Abbreviations

WD: Wellnessup diet
CRD: Calorie-restricted diet
MRD: Maintaining regular diet
BMI: Body mass index
LCD: Very low-calorie diet
POPs: Persistent organic pollutants
NCDs: Non-communicable diseases
WC: Waist circumference
HC: Hip circumference
WHR: Waist-to-hip ratio
MET: Metabolic equivalent task
