# Supplementary material for: Effect of toxic trace element detoxification, body fat reduction following four-week intake of the Wellnessup diet: a three-arm, randomized clinical trial
Source: Nutr Metab (Lond). 2020 Jun 22;17:47. doi: 10.1186/s12986-020-00465-9 (PMC7310262; doi:10.1186/s12986-020-00465-9)
Supplement: Supplementary file 2 — Additional file 2: Table S1. Provided mean dietary menu of test meals (per day) samples. Table S2. Changes in organic acid on urine. Table S3. Laboratory profiles of the participants in this study. [file 12986_2020_465_MOESM2_ESM.docx]

| **Supplementary Table 1-1**. Provided mean dietary menu of test meals (per day) samples | | | | | | | |
| --- | --- | --- | --- | --- | --- | --- | --- |
| **No.** | **WD (Wellness-up diet):** organic ingredients | | | | | | |
|  | **Breakfast** | **Lunch** | **Dinner** | | **Snack** | | **Total**  **(Kcal/d)** |
|  | **Shake(60g)** | **juices(450ml)** | **salads(400g)** | | **nuts bar(50g)** | |  |
| **1** | **A:**  Rice germ(20)  Rice bran(20)  Peapowder(10)  Black bean(8)  Blueberry extract(5)  Honey powder(5) | beat(60)  banana(100)  apple(145)  orange(145) | **tofu ricotta** | balsamic dressing | **B:**  Cashew nuts(5)  almond(7)  flaxseed(3)  sunflower seed(5)  chia seed(3) cranberry(4)  blue berry(4) brown rice bran(10)  Angelica gigas Nakai extract(7) | |  |
|  |  |  | oat(5) chickpea(10)  tofu(45)  lettuce(30) almond(10)  red cabbage(20)  onion(20)  ricota cheese(45) kale(15)  flower kale(15) cherry tomato(40) paprika(20)  walnut(5) | balsamic vinegar(10)  balsamic  glaze(10)  olive oil(10)  honey(10)  garlic(5)  sesame(1)  sesame oil(5) |  |  |  |
|  | 278.4 | 247.4 | 358.2 | 184.4 | 194.3 | | 1,259 |
| **2** | A | carrot(150)  ginger(1)  apple(245)  orange(245) | **curry chicken breast** | lemon dressing | B | |  |
|  |  |  | oat(5)  chicken breast(80) soy(10)  lettuce(30) almond(10)  red cabbage(20) onion(20)  kale(15)  flower kale(15) cherry tomato(40) paprica(20)  walnut(5) | honey(20)  lemon juice(7)  vinegar(10)  olive oil(10)  ginseng powder(3) |  |  |  |
|  | 278.4 | 296.5 | 329.7 | 167.5 | 194.3 | | 1,266 |
| **3** | A | lemon juice(10)  mango(45)  apple(160)  orange(160)  pineapple(150)  paprica(40) | **roasting tofu and mushroom** | black sesame seed dressing | B | |  |
|  |  |  | oat(5)  oyster mushroom(30) chickpea(10) tofu(75)  lettuce(30) almond(5)  red cabbage(20)  onion(15)  kale(15)  flower kale(15) cherry tomato(40)  shiitake mushrooms(30)  walnut(5) | soy sauce(5) honey(12)  mayonnaise(10) sugar(2)  salt(0.2) vinegar(5)  olive oil(10)  black sesame seed(7) |  |  |  |
|  | 278.4 | 230.3 | 222.3 | 245 | 194.3 | | 1,170 |
| **4** | A | apple(145)  lettuce(50)  orange(145)  kale(50)  kiwi(90) | **tofu ricotta** | balsamic dressing | | B |  |
|  |  |  | oat(5) chickpea(10)  tofu(45)  lettuce(30) almond(10)  red cabbage(20) onion(20)  ricota cheese(45) kale(15)  flower kale(15) cherry tomato(40) paprika(20)  walnut(5) | balsamic vinegar(10)  balsamic glaze(10)  olive oil(10)  honey(10)  garlic(5)  sesame(1)  sesame oil(5) | |  |  |
|  | 278.4 | 224.1 | 358.2 | 184.4 | 194.3 | | 1,240 |
| **5** | A | strawberry(25)  blueberry(100)  apple(137) cabbage(25)  orange(137)  campbell grapes(50) | **curry chicken breast** | lemon dressing | B | |  |
|  |  |  | oat(5)  chicken breast(80) soy(10)  lettuce(30) almond(10)  red cabbage(20) onion(20)  kale(15)  flower kale(15) cherry tomato(40) paprica(20)  walnut(5) | honey(20)  lemon juice(7) vinegar (10)  olive oil(10) ginseng powder(3) |  |  |  |
|  | 278.4 | 232.6 | 329.7 | 167.5 | 194.3 | | 1,203 |
| **Mean** | **278.4** | **246.2** | **509.4** | | **194.3** | | **1,228** |

*Continued*

**Supplementary Table 1-2**. Provided mean dietary menu of test meals (per day) samples

| **No.** | **CRD (calorie-restricted diet): common ingredients** | | | | | |
| --- | --- | --- | --- | --- | --- | --- |
|  | **Breakfast** | **Lunch** | **Dinner** | | **Snack** | **Total**  **(Kcal/d)** |
|  | **Shake(60g)** | **juices(450ml)** | **salads(400g)** | | **nuts bar(50g)** |  |
| **1** | **C:**  Yogurt powder (98.2%)  Vitamin E (1.7%)  Vitamin B12 (0.1%) | orange(300)  tomato(150) | **tofu ricotta** | **balsamic dressing** | **D:**  cashew nuts(5),almond(7)  flaxseed(3),  sunflower seed(5),  chia seed(3), cranberry(4)  blue berry(4), brown rice bran(10),  Angelica gigas Nakai extract(7) |  |
|  |  |  | oat(5) chickpea(10), tofu(45), lettuce(30), almond(10),  red cabbage(20),  onion(20),  ricota cheese(45), kale(15),  flower kale(15), cherry tomato(40), paprika(20), walnut(5) | balsamic vinegar(10), balsamic glaze(10), olive oil(10), honey(10), garlic(5), sesame(1), sesame oil(5) |  |  |
|  | 245 | 267 | 358.2 | 184.4 | 194.3 | 1,249 |
| **2** | C | orange(300)  tomato(150) | **curry chicken breast** | lemon dressing | D |  |
|  |  |  | oat(5),  chicken breast(80), soy(10), lettuce(30), almond(10),  red cabbage(20), onion(20), kale(15), flower kale(15), cherry tomato(40), paprica(20), walnut(5) | honey(20),  lemon juice(7), vinegar (10),  olive oil(10), ginseng powder(3) |  |  |
|  | 245 | 267 | 329.7 | 167.5 | 194.3 | 1,204 |
| **3** | C | orange(300)  tomato(150) | **roasting tofu and mushroom** | black sesame seed dressing | D |  |
|  |  |  | oat(5), oyster mushroom(30), chickpea(10), tofu(75), lettuce(30), almond(5), red cabbage(20), onion(15), kale(15), flower kale(15), cherry tomato(40),  shiitake mushrooms(30), walnut(5) | soy sauce(5) honey(12)  mayonnaise(10), sugar(2), salt(0.2) vinegar(5),  olive oil(10),  black sesame seed(7) |  |  |
|  | 245 | 267 | 222.3 | 245 | 194.3 | 1,174 |
| **4** | C | orange(300)  tomato(150) | **tofu ricotta** | balsamic dressing | D |  |
|  |  |  | oat(5) chickpea(10), tofu(45), lettuce(30), almond(10),  red cabbage(20), onion(20),  ricota cheese(45), kale(15),  flower kale(15), cherry tomato(40), paprika(20), walnut(5) | balsamic vinegar(10), balsamic glaze(10),  olive oil(10), honey(10), garlic(5), sesame(1), sesame oil(5) |  |  |
|  | 245 | 267 | 358.2 | 184.4 | 194.3 | 1,249 |
| **5** | C | orange(300)  tomato(150) | **curry chicken breast** | lemon dressing | D |  |
|  |  |  | oat(5),  chicken breast(80), soy(10), lettuce(30), almond(10),  red cabbage(20), onion(20), kale(15), flower kale(15), cherry tomato(40), paprica(20), walnut(5) | honey(20),  lemon juice(7), vinegar (10),  olive oil(10), ginseng powder(3) |  |  |
|  | 245 | 267 | 329.7 | 167.5 | 194.3 | 1,204 |
| **Mean** | **245** | **267** | **509.4** | | **194.3** | **1,216** |


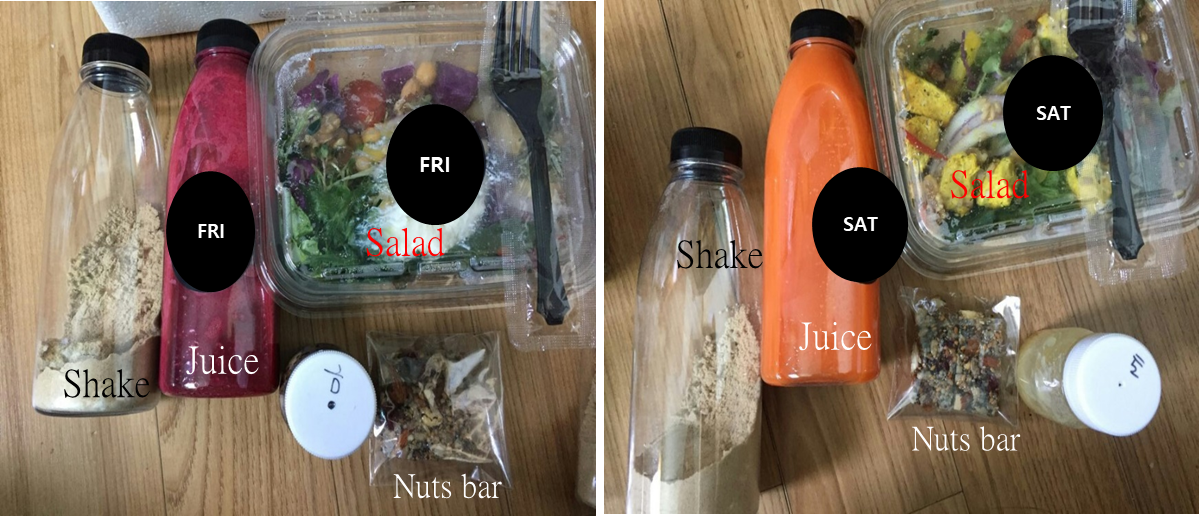


< **Samples of provided test meals by week >**

| **Supplementary Table 2.** Changes in organic acid on urine | | | | | | | | |
| --- | --- | --- | --- | --- | --- | --- | --- | --- |
| **Variables** | **WD**  **(n=15)** | **CRD**  **(n=15)** | **MRD**  **(n=15)** | **p-value^2)^ (W-M-C)** | | **p-value^3)^ (W-M)** | **p-value^4)^ (W-C)** | **p-value^5)^ (M-C)** |
| **β-Hydroxybutyrate** (0~11.9 mmol/mol Cr) | | | | | | | | |
| Baseline | 0.90(0.62-1.96) | 0.91(0.71-3.15) | 1.08(0.55-1.49) |  |  | |  |  |
| 4-week | 1.26(0.75-4.46) | 6.27(2.96-12.3) | 0.80(0.56-1.87) | 0.174 | 0.184 | | 0.740 | 0.075 |
| p-value^1)^ | 0.208 | **0.003** | 0.421 |  |  | |  |  |
| **Isocitrate** (23~84.2 mmol/mol Cr) | | | | | | | | |
| Baseline | 25.1(23.1-27.0) | 32.8(23.6-36.8) | 27.8(22.9-30.8) |  |  | |  |  |
| 4-week | 32.1(27.4-37.1) | 32.1(28.4-38.4) | 28.4(22.7-33.8) | 0.564 | 0.590 | | 0.300 | 0.619 |
| p-value | **0.041** | 0.135 | 0.600 |  |  | |  |  |
| **Methylmalonate** (0~2.3 mmol/mol Cr) | | | | | | | | |
| Baseline | 1.14(0.90-1.51) | 1.04(0.88-1.51) | 1.36(0.83-2.13) |  |  | |  |  |
| 4-week | 0.89(0.51-1.26) | 1.00(0.70-1.33) | 0.99(0.83-1.54) | 0.279 | 0.115 | | 0.455 | 0.431 |
| p-value | 0.359 | **0.002** | **0.041** |  |  | |  |  |
| **α-Ketoisocaproate** (0~0.45 mmol/mol Cr) | | | | | | | | |
| Baseline | 0.17(0.14-0.19) | 0.18(0.16-0.20) | 0.17(0.15-0.22) |  |  | |  |  |
| 4-week | 0.19(0.16-0.22) | 0.22(0.19-0.28) | 0.17(0.15-0.23) | **0.050** | **0.028** | | **0.050** | 0.868 |
| p-value | **0.001** | **0.229** | 0.208 |  |  | |  |  |
| **α-Hydroxybutyrate** (0~1.8 mmol/mol Cr) | | | | | | | | |
| Baseline | 0.35(0.21-0.40) | 0.36(0.21-0.64) | 0.30(0.20-0.47) |  |  | |  |  |
| 4-week | 0.52(0.31-0.93) | 0.65(0.39-1.14) | 0.35(0.19-0.82) | 0.553 | 0.772 | | 0.648 | 0.229 |
| p-value | **0.010** | **0.001** | **0.035** |  |  | |  |  |
| **8-Hydroxy-2-deoxyguanosine** (0~11.4 ng/mg Cr) | | | | | | | | |
| Baseline | 2.34(1.90-2.97) | 2.70(2.13-3.82) | 2.43(1.92-2.86) |  |  | |  |  |
| 4-week | 2.14(1.06-2.97) | 2.20(1.66-2.51) | 1.52(0.94-2.03) | 0.272 | 0.199 | | 0.901 | 0.147 |
| p-value | **0.050** | **0.009** | 0.473 |  |  | |  |  |
| Values are presented as median (interquartile range)  ^1)^ Analyzed by the Wilcoxon signed-rank test  ^2)^ Analyzed by the Kruskal Wallis test for WD-CRD-MRD groups . ^3)^ Analyzed by the Wilcoxon rank-sum test for change in WD-MRD groups. ^4)^ Analyzed by the Wilcoxon rank-sum test for change in WD-CRD groups. ^5)^ Analyzed by the Wilcoxon rank-sum test for change in MRD-CRD groups. Abbreviations: WD, Wellnessup diet; MRD, maintaining regular diet; CRD, calorie-restricted diet. | | | | | | | | |

| **Supplementary Table 3.** Laboratory profiles of the participants in this study | | | | | | | |
| --- | --- | --- | --- | --- | --- | --- | --- |
| **Laboratory profiles (standard range)** | **WD** **(n=15)** | | **CRD (n=15)** | | **MRD** **(n=15)** | |  |
|  | **Week 0** | **Week 4** | **Week 0** | **Week 4** | **Week 0** | **Week 4** | **p-value^1^** |
| WBC (4.8–10.8×10^3^ /μL) | 6.49±1.90 | 5.89±2.45 | 5.91±1.28 | 5.15±1.39 | 6.29±1.69 | 6.00±1.58 | 0.442 |
| RBC (4.2–5.4×100^3^ /μL) | 4.46±0.28 | 4.35±0.30 | 4.40±0.33 | 4.66±0.33 | 4.51±0.22 | 4.52±0.29 | 0.005 |
| Hemoglobin (12–16 g/dL) | 13.46±0.83 | 13.37±0.91 | 12.96±0.91 | 13.59±1.06 | 13.16±0.98 | 13.16±1.04 | 0.001 |
| Hematocrit (37–47%) | 40.45±2.42 | 40.48±2.65 | 38.952±2.47 | 40.92±2.66 | 39.61±2.16 | 39.66±2.50 | 0.007 |
| Platelet (130–450×10^3^ /μL) | 280.33±45.44 | 269.67±58.21 | 265.27±68.22 | 248.73±63.37 | 291.67±58.54 | 295.20±70.65 | 0.118 |
| GGT (8–48 IU/L) | 29.67±49.54 | 22.00±31.92 | 9.93±4.77 | 8.80±4.25 | 17.07±8.90 | 15.27±8.47 | 0.435 |
| AST (12–33 IU/L) | 20.40±7.93 | 22.40±7.61 | 21.93±9.12 | 20.53±4.07 | 18.27±2.66 | 19.93±4.01 | 0.227 |
| ALT (5–35 IU/L) | 19.93±12.80 | 22.40±15.21 | 18.13±9.91 | 16.80±8.24 | 17.73±5.39 | 18.20±4.78 | 0.196 |
| Total bilirubin (0.2–1.2 mg/dL) | 0.66±0.33 | 0.83±0.21 | 0.75±0.28 | 1.07±0.34 | 0.68±0.15 | 0.78±0.22 | 0.128 |
| Total protein (6.7–8.3 g/dL) | 7.55±0.46 | 7.80±0.35 | 7.59±0.33 | 7.95±0.28 | 7.62±0.29 | 7.82±0.36 | 0.586 |
| Albumin (3.5–5.3 g/dL) | 4.64±0.24 | 4.57±0.19 | 4.57±0.19 | 4.86±0.15 | 4.63±0.26 | 4.75±0.26 | 0.169 |
| BUN (8–23 mg/dL) | 12.87±2.50 | 11.47±2.10 | 11.20±2.11 | 11.87±3.00 | 11.80±2.73 | 12.53±3.36 | 0.076 |
| Creatinine (0.7–1.7 mg/dL) | 0.63±0.08 | 0.67±0.07 | 0.61±0.09 | 0.65±0.10 | 0.59±0.09 | 0.61±0.08 | 0.782 |
| Total cholesterol (mg/Dl) | 196.93±32.84 | 182.33±27.66 | 182.13±29.34 | 170.87±27.92 | 207.27±038.31 | 201.93±33.53 | 0.516 |
| Apo A1 (g/dL) | 1.61±0.30 | 1.57±0.26 | 1.60±0.21 | 1.51±0.16 | 1.68±0.25 | 1.62±0.22 | 0.629 |
| Apo B (g/dL) | 0.97±0.21 | 0.88±0.16 | 0.85±0.18 | 0.79±0.16 | 0.99±0.27 | 0.96±0.24 | 0.622 |
| Triglyceride (mg/dL) | 96.13±44.13 | 86.53±41.14 | 74.07±27.14 | 66.87±24.09 | 107.20±64.91 | 101.67±52.75 | 0.914 |
| HDL-cholesterol (mg/dL) | 63.73±14.10 | 62.67±12.29 | 63.47±13.23 | 61.27±10.03 | 67.87±17.08 | 65.67±14.43 | 0.905 |
| LDL-cholesterol (mg/dL) | 108.27±27.83 | 94.73±25.19 | 96.93±26.42 | 86.27±24.58 | 113.40±33.87 | 105.93±28.23 | 0.712 |
| Uric acid(mg/Dll) | 5.21±1.08 | 5.61±0.97 | 4.74±1.06 | 5.29±1.24 | 5.13±0.82 | 5.55±0.93 | 0.957 |
| Glucose (74–106 mg/dL) | 92.27±13.51 | 84.33±8.56 | 89.40±7.38 | 82.53±7.38 | 88.33±6.02 | 82.33±9.08 | 0.915 |
| Insulin (μU/mL) | 11.80±4.49 | 10.56±3.92 | 9.29±4.13 | 8.43±3.31 | 12.14±8.46 | 11.68±9.25 | 0.780 |
| HbA1c (%) | 5.41±0.21 | 5.40±0.17 | 5.29±0.21 | 5.29±0.21 | 5.37±0.36 | 5.41±0.39 | 0.843 |
| hs-CRP (mg/L) | 1.80±2.99 | 1.27±1.85 | 0.96±1.87 | 0.41±0.773 | 0.95±1.48 | 1.50±1.93 | 0.374 |
| Creatinine Kinase (IU/L) | 119.87±181.55 | 102.00±87.00 | 184.33±399.92 | 105.73±97.32 | 73.87±20.65 | 120.53±106.76 | 0.180 |
| Specific Gravity | 1.03±0.01 | 1.03±0.01 | 1.02±0.01 | 1.03±0.01 | 1.02±0.01 | 1.03±0.000 | 0.669 |
| pH | 5.87±0.48 | 6.10±0.71 | 6.07±0.84 | 5.97±0.48 | 6.13±0.81 | 5.93±0.68 | 0.176 |
| Data are presented as mean ± SD.  ^1^Analyzed by repeated measure analysis of variance; p-value indicates significant differences between treatment groups from baseline.  Abbreviations: WD, Wellnessup diet; MRD, maintaining regular diet; CRD, calorie-restricted diet. | | | | | | | |
